# *α*-Bisabolol Mitigates Colon Inflammation by Stimulating Colon PPAR-*γ* Transcription Factor: In Vivo and In Vitro Study

**DOI:** 10.1155/2022/5498115

**Published:** 2022-04-13

**Authors:** Balaji Venkataraman, Saeeda Almarzooqi, Vishnu Raj, Pradeep K. Dudeja, Bhoomendra A. Bhongade, Rajesh B. Patil, Shreesh K. Ojha, Samir Attoub, Thomas E. Adrian, Sandeep B. Subramanya

**Affiliations:** ^1^Department of Physiology, College of Medicine and Health Sciences, United Arab Emirates University, PO Box—17666, Al Ain, UAE; ^2^Zayed Bin Sultan Center for Health Sciences, College of Medicine and Health Sciences, United Arab Emirates University, PO Box—17666, Al Ain, UAE; ^3^Department of Pathology, College of Medicine and Health Sciences, United Arab Emirates University, PO Box—17666, Al Ain, UAE; ^4^Jesse Brown Veterans Affairs Medical Center, Chicago, Illinois, USA; ^5^Division of Gastroenterology and Hepatology, Department of Medicine, University of Illinois at Chicago, Chicago, Illinois, USA; ^6^Department of Pharmaceutical Chemistry, RAK College of Pharmaceutical Chemistry, RAK Medical & Health Sciences University, Ras Al Khaimah, UAE; ^7^Department of Pharmaceutical Chemistry, STES's Smt. Kashibai Navale College of Pharmacy, Kondhwa (Bk), Pune, India; ^8^Department of Pharmacology and Therapeutics, College of Medicine and Health Sciences, United Arab Emirates University, PO Box—17666, Al Ain, UAE; ^9^Department of Basic Medical Sciences, College of Medicine, Mohammed Bin Rashid University of Medicine and Health Sciences, PO Box—505055, Dubai, UAE

## Abstract

The incidence and prevalence of inflammatory bowel disease (IBD, Crohn's disease, and ulcerative colitis) are increasing worldwide. The etiology of IBD is multifactorial, including genetic predisposition, dysregulated immune response, microbial dysbiosis, and environmental factors. However, many of the existing therapies are associated with marked side effects. Therefore, the development of new drugs for IBD treatment is an important area of investigation. Here, we investigated the anti-inflammatory effects of *α*-bisabolol, a naturally occurring monocyclic sesquiterpene alcohol present in many aromatic plants, in colonic inflammation. To address this, we used molecular docking and dynamic studies to understand how *α*-bisabolol interacts with PPAR-*γ*, which is highly expressed in the colonic epithelium: in vivo (mice) and in vitro (RAW264.7 macrophages and HT-29 colonic adenocarcinoma cells) models. The molecular docking and dynamic analysis revealed that *α*-bisabolol interacts with PPAR-*γ*, a nuclear receptor protein that is highly expressed in the colon epithelium. Treatment with *α*-bisabolol in DSS-administered mice significantly reduced Disease Activity Index (DAI), myeloperoxidase (MPO) activity, and colonic length and protected the microarchitecture of the colon. *α*-Bisabolol treatment also reduced the expression of proinflammatory cytokines (IL-6, IL1*β*, TNF-*α*, and IL-17A) at the protein and mRNA levels. The expression of COX-2 and iNOS inflammatory mediators were reduced along with tissue nitrite levels. Furthermore, *α*-bisabolol decreased the phosphorylation of activated mitogen-activated protein kinase (MAPK) signaling and nuclear factor kappa B (NF*κ*B) proteins and enhanced colon epithelial PPAR-*γ* transcription factor expression. However, the PPAR-*α* and *β*/*δ* expression was not altered, indicating *α*-bisabolol is a specific stimulator of PPAR-*γ*. *α*-Bisabolol also increased the PPAR-*γ* transcription factor expression but not PPAR-*α* and *β*/*δ* in pretreated in LPS-stimulated RAW264.7 macrophages. *α*-Bisabolol significantly decreased the expression of proinflammatory chemokines (CXCL-1 and IL-8) mRNA in HT-29 cells treated with TNF-*α* and HT-29 PPAR-*γ* promoter activity. These results demonstrate that *α*-bisabolol mitigates colonic inflammation by inhibiting MAPK signaling and stimulating PPAR-*γ* expression.

## 1. Introduction

Inflammatory bowel diseases (IBD) are chronic inflammatory disorders that include Crohn disease (CD) and ulcerative colitis (UC), affecting the entire gastrointestinal tract or restricted to the colon. These disorders are characterized by relapsing and remitting intestinal inflammation with epithelial damage. The incidence and prevalence of IBD are rising globally [1, 2]. The etiology of IBD is multifactorial, including genetic susceptibility, dysregulated immune response, and gut microbiome dysregulation, as well as environmental factors. Drugs used for IBD therapy have limitations due to various factors existing in individual IBD patients. Therefore, the development of new drugs to treat IBD patients continues to embody significant areas of investigation.

Peroxisome proliferator-activated receptors (PPARs), a cluster of nuclear receptor proteins, regulate lipid metabolism, cell proliferation, and insulin sensitization and play a critical role in limiting intestinal inflammation [3]. There are three isoforms of PPARs, PPAR*α*, PPAR*β*/*δ*, and PPAR-*γ*. PPAR-*γ* is expressed in three forms PPAR-*γ* 1, 2, and 3 that are produced from the same gene by alternative splicing. Interestingly, PPAR-*γ* 1 and PPAR-*γ* 3 are codes for a 475 amino acid identical protein [4]. PPAR-*γ* 1 is ubiquitously expressed, while PPAR-*γ* 3 is only highly expressed in the colon, adipose tissue, and macrophages [4]. Indeed, the colonic muscosa has the highest expression of PPAR-*γ* of any tissue in the body [5]. PPARs heterodimerize with retinoid X receptors (RXR), and this complex then binds to specific regions of DNA called the PPAR response elements (PPRE) to activate transcription of target genes. The anti-inflammatory response of PPARs is mediated by inhibiting production of proinflammatory cytokine and chemokines when activated either by endogenous, synthetic, or phytochemical ligands [6–8]. In particular, PPAR-*γ* 1 and PPAR-*γ* 3 are highly expressed in the colon epithelium and to a lesser extent in macrophages and lymphocytes [9]. The PPAR-*γ* expression is downregulated up to 60% at mRNA and protein level in the colonic mucosa of UC patients compared to CD patients [9]. Thus, modulating PPAR-*γ* in the colon becomes a potential drug target that can be exploited for the treatment of inflammatory bowel disease, particularly ulcerative colitis, as well as colon cancer.

*α*-Bisabolol is a natural monocyclic sesquiterpene alcohol, found in Matricaria chamomilla and other aromatic plants such as Eremanthuserythropappus, Smyrniopsisaucheri, Salvia runcinata, and Vanillosmopsis species [10, 11] shown to possess potent analgesic, antimicrobial, and anti-inflammatory properties [12–14]. In a murine model of osteoarthritis, *α*-bisabolol suppressed the inflammation and extracellular matrix (ECM) degeneration induced by advanced glycation end products (AGE) [15]. *α*-Bisabolol cotreatment in LPS-stimulated RAW264.7 macrophages inhibited the expression of proinflammatory mediators such as COX-2, iNOS expression, and the NF-*κ*B signaling pathway to reduce the proinflammatory cytokine response [14]. Activation of NF-*κ*B plays an important role in the transcription of proinflammatory cytokines. Overt activation of NF-*κ*Bsignaling is observed in the macrophages and the epithelial cells isolated from the IBD patients concurring with the severity of inflammation in these patients [16].

In silico docking analysis revealed that *α*-bisabolol has a strong binding affinity for the PPAR-*γ* binding site. PPAR-*γ* forms a complex with the NF-*κ*B subunit p65 at a nuclear level leading to the alteration of proinflammatory gene expression mediated by NF-*κ*B. Inhibition of NF-*κ*B in response to the activity of PPAR-*γ* ligands attenuates the expression of various cytokines in colonic epithelial cells such as IL-1*β*, COX-2, IL-6, IL-8, TNF-*α*, IFN*γ*, iNOS, and chemokines [9, 17]. Based on these observations, we investigated the molecular mechanisms mediating the anti-inflammatory effects of *α*-bisabolol in a murine model of colon inflammation, LPS-stimulated RAW264.7 macrophages, and TNF-*α*-challenged colonic adenocarcinoma (HT-29) cells.

## 2. Materials and Methods

### 2.1. Chemical, Reagents, and Cells

There is dextran sulfate sodium (DSS) (MW 36,000–50,000 kDa-MP Biomedicals, Solon, OH, USA). *α*-Bisabolol, hexadecyltrimethylammonium bromide (HTAB), and orthodianisidine dihydrochloride (ODD) were purchased from Sigma-Aldrich (St. Louis, MO, USA). IL-6, IL-1*β*, and TNF-*α* ELISA kits were purchased from R&D systems (Minneapolis, MN, USA). The reverse transcription kit was procured from Applied Biosystems (Foster City, CA, USA). There is EvaGreen 5× mastermix from Solis BioDyne (Tartu, Estonia). Macrogen Inc. (Seoul, South Korea) provided the mastermix and primers for quantitative RT-PCR. The protease and phosphatase inhibitor were procured from Thermo-Scientific (Rockford, IL, USA). Antibodies were purchased from Santa Cruz Biotechnology (Dallas, TX, USA); catalogue numbers were given in our precious publication [16]. Commercially available pNL 1.3 and pNL1.3. CMV vectors were purchased from Promega (Cat# N1021 & N1101, Madison, WI, USA), and the PPRE-pNL1.3 plasmid was purchased from Addgene (Cat#84394, Watertown, MA, USA). Other reagents were obtained from the suppliers listed in our previous publication [18].

The RAW264.7 macrophages and HT-29 colorectal adenocarcinoma cells were obtained from the American Type Culture Collection (Manassas, VA, USA).

### 2.2. Molecular Docking

A molecular docking study was performed on the PPAR-*γ* ligand-binding domain. The crystal structure PPAR-*γ* ligand-binding domain (PDB ID: 5Y2T) resolved at 1.70 Å was retrieved from a protein databank (http://www.rcsb.com). While preparing the protein structure for docking simulation, the bound ligand and the water molecules were removed. Further, hydrogen atoms were added and subsequently, the positions of added hydrogen atoms were optimized with a gradient norm of 0.05 in the Tinker 8 program [19]. The correct protonation states to each residue were assigned using the PROPKA program (citation: doi:10.1021/ct200133y). The 2D structures of bound ligand (lobeglitazone) and *α*-bisabolol were drawn and subsequently transformed to 3D structures in the Marvin Sketch 5.6.0.0 program (http://www.chemaxon.com). The resulting 3D structures were geometry optimized after assigning gasteiger charges in the UCSF Chimera 1.8 [20] program with the combination of the steepest descent and conjugate gradient geometry search criteria until gradient converges to 0.05 and 0.01, respectively. Molecular docking studies were performed with Autodock Vina [21]. To capture the binding site adaptability of the PPAR-*γ* binding site, the residues forming hydrogen bond interactions with bound lobeglitazone, namely, Ser289, His323, His449, and Tyr473, and residues forming hydrophobic interactions, namely, Cys285, Leu330, Ile341, and Met348 were picked as flexible residues. The grid box was set around the center of the bound ligand having dimensions 22 × 24 × 26 Å, large enough to search for the binding site's possible conformational space. Flexible docking was performed with exhaustiveness of 100 to access the entire search space of the binding site and the resultant binding free energy estimates, binding poses, and key interactions analyzed.

### 2.3. Molecular Dynamic Simulation Studies and MM-PBSA Calculations

The PPAR-*γ* bare protein, as well as the docked complex of *α*-bisabolol at the binding site of PPAR-*γ*, was subjected to the MD simulations using Gromacs 4.5.6 [22]. The MD simulations were performed on a remote server of the Bioinformatics Resources and Applications Facility (BRAF), C-DAC, Pune. The topology of the protein was constructed with the parameters implemented in the Gromos54a7 force field [23] while the ligand topologies compatible with Gromos54A7 were generated in ATB server [24, 25]. To properly solvate the system, a simple point charge (SPC216) water model [26] was used, and the system was neutralized with appropriate ions such as sodium and chloride. This solvated system was subjected to unrestrained energy minimization with the steepest descent criteria to remove the steric clashes. The system was equilibrated at constant pressure, volume, and temperature of 300 K conditions for 100 picoseconds. This equilibrated system was subjected to 50 ns MD simulation with retraining on covalent bonds using the LINCS algorithm [27] and the cut-off value of 12 Å for the long-range electrostatics such as Coulomb and Lennard Jones with the particle mesh Ewald method [28]. Post-MD analysis was carried out through measurement of deviations in the protein and ligand atoms as root mean square deviations (RMSD). The extent of fluctuations in the atoms of residues in protein in terms of root means square fluctuations (RMSF) measurement was also considered in estimating the stability of protein-ligand complexes. The radius of gyration (Rg), a root mean square distance of a collection of atoms from their common center of mass and which provides the information of compactness of protein structure, was also analyzed. Hydrogen bond formation is one of the most important non-bonded interactions. The phenomenon of hydrogen bond formation during the progress of MDS was analyzed along with the number of hydrogen bonds formed.

### 2.4. Animals

C57BL/6 J mice (12 weeks old) weighing 25–30 g were procured from central animal facility, CMHS, UAE University. Two animals per cage were kept for each experiment 1 week before the start of the experiment for acclimatization. The animals were maintained at temperature 23 ± 1°C, a 12 h light-dark cycle, with 50–60% humidity. Food and water were provided ad libitum. The UAEU Institutional Animal Ethical committee has approved the current study (approval # ERA_2019_5845).

### 2.5. Experimental Design

Mice were randomly allocated to 5 groups with 8 animals in each group): group I: untreated control. Group II: DSS alone. Group III: DSS + bisabolol (Bis) (100 mg/kg body weight/day). Group IV: DSS + Bis (200 mg/kg body weight/day). Group V: DSS + SAZ (50 mg/kg body weight/day). DSS (2%) was prepared freshly every day in autoclaved drinking water. At the end of 8-day treatment protocol, the animals were euthanized using pentobarbital overdose (100 mg/kg body weight). Colons were surgically removed and length measured for DAI, including the caecum. Colons were then cleaned with ice-cold saline to remove fecal content. After cleaning, the colon was scraped to separate mucus layer, and those were snap-frozen immediately using liquid nitrogen and stored at −80°C until further use. Another set of colons were cut longitudinally and coiling started from proximal (in the center) to distal as like swiss roll and then fixed using 10% formalin for hematoxylin and eosin (H&E) staining.

### 2.6. Evaluation of Disease Activity Index (DAI) Score

Mice were weighed daily and observed for loose stools presence, diarrhea, and bleeding. The DAI scores calculated were based on parameters as published in our previous study [29]. In short, the DAI scores comprise of the scores acquired from daily recording of loss of weight, loose stools/diarrhea, and bleeding as indicated in Table 1.

### 2.7. Proinflammatory Cytokine Estimation by Enzyme-Linked Immunosorbent Assay (ELISA)

Colonic mucosa was homogenized with phosphatase and protease inhibitor cocktail tablet (cat#A32959, Thermo-Scientific, USA) dissolved in RIPA buffer (Cat# 20-188, Millipore, St. Louis, MO, USA) with zirconium beads (2 mm, Cat# 11079124zx, Biospec, Bartlesville, OK, USA) in Precellys 24 tissue homogenizer (Bertin Instruments, Montigny-le-Bretonneux, France). Then, the resultant homogenate was centrifuged at 1000 × g at 4°C. After centrifugation, the supernatant was transferred into a fresh microcentrifuge tube with gentle mixing on the tube rotator overnight at 4°C. The homogenate was centrifuged at 15,000 × g at 4°C for 30 min, the supernatant was diluted (1 : 3) with RIPA buffer, and its protein concentration was estimated using Pierce BCA Protein Assay Kit (Cat# 23225, Thermo-Scientific, USA). Undiluted homogenate was kept in aliquots at -80°C for later use. TNF-*α*, IL-1*β*, IL-6, and IL-17 cytokines were determined by ELISA assay in the colonic mucosal homogenates according to the manufacturer's instructions.

### 2.8. Myeloperoxidase (MPO) Assay

Tissue MPO activity was measured as described previously [30]. Briefly, ~25 mg freshly scraped colonic mucosa was homogenized using zirconium beads in 50 mM phosphate buffer (pH 6) containing 0.5% hexadecyltrimethylammonium bromide (HTAB). Homogenates were processed through a freeze-thaw cycle (liquid nitrogen and a 25°C water bath) and sonicated for 30 sec. This process was repeated three times. Suspensions were then centrifuged at 20,000 g for 20 min at 4°C. The supernatant (0.1 mL) was added with 2.9 mL of in 50 mM phosphate buffer (pH 6) containing 0.53 mM of o-dianisidine hydrochloride and 0.15 mM hydrogen peroxide, and the change in absorbance was measured every 15 s for, 5 min at 460 nm. These results were expressed in units (U) of MPO/mg of protein. The protein concentration of supernatants was determined by Pierce BCA Protein Assay Kit (Pierce, Rockford, IL, USA). MPO activity was determined as mean absorbance at 460 nm/incubation time/protein concentration.

### 2.9. Histopathological Evaluation

The prepared swiss roll-colon was fixed using 10% formaldehyde solution overnight. The dehydration process was carried out using ethanol in increasing order of concentration. The dehydrated tissues were embedded in paraffin. This paraffin embedded (swiss roll) colon was cut into thin slice (2 *μ*m thick) and was stained using H&E for the histological analysis. To evaluate the changes in histopathology, a clinical pathologist determined each sample's score. These samples were blinded for histopathological evaluation: inflammation score = grade of inflammation × percentage of involvement. The colonic inflammation degree was determined as per previously published study explained in Table 2 [31].

### 2.10. RNA Extraction and Real-Time RT-PCR

The RNA extraction from colonic mucosa and conversion in to cDNA and real-time PCR was performed as described previously [32]. The 18 s gene product was used as an internal reference gene in the present study. The change in CT values was calculated using the delta CT method CT (2-*ΔΔ*CT) [33]. Primer sequences for all genes used in the present study were reported in our previous study [32]. Primers used for real-time PCR analysis as follows: human PPAR-*γ* (PMID No. 20421464): forward: 5′-TTCAAGAGTACCAAAGTGCAATCAA-3′, reverse: 5′-AATAAGGTGGAGATGCAGGCTC-3′. Human PPAR*α* (PMID No. 28732066): forward: 5′-ATCGGCGAGGATAGTTCT-3′, reverse: 5′-AATCGCGTTGTGTGACAT-3′. Human PPAR*β*/*δ* (PMID No. 16197558): forward: 5′-GTCACACAACGCTATCCGTTT-3′, reverse: 5′-AGGCATTGTAGATGTGCTTGG-3′. Mouse PPAR*α* (PMID No. 26308623): forward: 5′-ATGCCAGTACTGCCGTTTTC-3′, reverse: 5′-CCGAATCTTTCAGGTCGTGT-3′. Mouse PPAR*β*/*δ* (PMID No. 28404991): forward: 5′-CTCAATGGGGGACCAGAACA-3′, reverse: 5′-AAGGGGAGGAATTCTGGGAGA-3′. Mouse IL-17A (PMID No. 27578011): forward: 5′-ATCCCTCAAAGCTCAGCGTGTC-3′, reverse: 5′-GGGTCTTCATTGCGGTGGAGAG-3′.

### 2.11. Western Blot

Colonic mucosa frozen samples were homogenized in RIPA buffer with protease and phosphatase inhibitors cocktail using a bead homogenizer as previously described [32]. The undiluted homogenate protein concentrations were determined using Pierce BCA Protein Assay Kit (Cat# 23225, Thermo-Scientific, USA). 20 *μ*g (colon tissue, and RAW264.7 macrophages) proteins were resolved using sodium dodecyl sulfate-polyacrylamide gel electrophoresis (SDS-PAGE) using 8-12% gels and subsequently transferred onto PVDF membrane. These PVDF membranes were immune blotted using specific antibodies (COX-2, iNOS, PPAR-*α*, PPAR-*β*/*δ*, PPAR-*γ*, p^Ser536^NF*κ*B p65). The internal control used to normalize the blots was GAPDH.

### 2.12. Measurement of Superoxide Dismutase, Catalase Enzyme Activity, and Tissue Nitrite Concentration

Superoxide dismutase (SOD) was assayed by using the method of Kakkar et al. [34] based on 50% inhibition of the formation of NADH–phenazine methosulphate–nitroblue tetrazolium (NBT) formazan at 520 nm. One unit of the enzyme taken as the amount of enzyme was required for 50% inhibition of NBT reduction/min/mg protein. Catalase activity (CAT) was determined by the method of Sinha [35]. The values of CAT activity were expressed as moles of H_2_O_2_ utilized/min/mg protein. The levels of nitric oxide (NO) in colon homogenate were measured using a Griess reagent by the method of Lu et al. [36]; nitrite concentration, an indicator of NO production, was calculated from a NaNO_2_ standard curve and expressed as *μ* moles/mg protein. Snap frozen colon samples were processed for biochemical estimation on the following day of sample collection.

### 2.13. RAW Macrophages and HT-29 Cell Culture

RAW264.7 macrophages and HT-29 colon cancer cells were cultured at 37°C and 5% CO_2_ in a humidified incubator in high-glucose DMEM, containing 100 U/mL penicillin, 100 *μ*g/mL streptomycin, and 10% (v/v) heat-inactivated fetal bovine serum (FBS). Cultured macrophages were seeded (1.5 × 10^5^ cells per well) onto 6-well plates 24 h before treatment. To induce inflammation, the macrophages were treated with 1 mg/mL LPS with or without *α*-bisabolol (20 and 40 *μ*M) for 24 h. After treatment, medium was collected for the expression of inflammatory mediators (IL-6, IL-1*β*, TNF-*α*), and cells were collected with RIPA buffer added with protease inhibitors for measurement of PPAR-*α*, PPAR-*β*/*δ*, PPAR-*γ*, and p^Ser536^NF*κ*B p65 proteins. HT-29 cells were used to perform the PPAR-*γ* promotor assay. HT-29 cells were utilized only for PPAR-*γ* promotor/nanoluciferase assay.

### 2.14. Cell Viability Assay

Macrophage and HT-29 cells (5000 cells/well) were seeded onto 96-well plates and treated with a range of *α*-bisabolol concentrations (0, 12.5, 50, 100, 150, and 200 *μ*M) for 24 h and 48 h. According to the manufacturer's instructions, cell viability was determined using the Cell Titer-Glo® viability kit after the indicated treatment period. Luminescence was measured using a Tecan (Infinite® 200 PRO) plate reader. Data are represented as percent of viable cells (quantified by ATP content) in the *α*-bisabolol treated groups compared to the untreated, control group.

### 2.15. PPAR-*γ* Promotor andNanoluciferase Assay

The vectors and plasmid (expressing lumniscentNanoLuc® luciferase) were transformed into JM109 competent bacterial cells by the ligation method and cultured overnight in 125 mL media at 37°C. The plasmid was extracted using the Plasmid Purification Maxiprep Kit (Cat# 12162, Qiagen, Germany) following the manufacturers' instructions. The plasmid DNA and Lipofectamine LTX mixture wer prepared in serum and antibiotic-free DMEM media as described previously [18]. The transient transfection with PPRE-pNL1.3 or pNL1.3 basic secreted luciferase reporter used as control. HT-29 cells were transfected in 24-well plates (Cat# 3516, Corning Costar, Kennebunk, ME, USA) using Lipofectamine LTX Plus (Invitrogen, Carlsbad, CA, USA) following the manufacturer's protocol. After 24 hours, Lipofectamine LTX Plus added media were removed, replaced with fresh 2% FBS-media containing *α*-bisabolol 40 *μ*M or PPAR-*γ* agonist (GW1929) or PPAR-*γ* antagonist (GW9662) or GW9662 + *α*-bisabolol 40 *μ*M for 24 h period. After 24 hr treatment, each cell supernatant was dispensed into 96-well black plate (Cat# 237105, Thermo-Scientific, Roskilde, Denmark), and the secreted NanoLuc luciferase activity was determined using the Nano-Glo® Luciferase assay buffer (Cat# N1120, Promega, Madison, WI, USA) according to the manufacturer's instructions. Luminescence in each well was then measured by using a Tecan Multimode plate reader.

### 2.16. Statistical Analysis

Statistical analysis was performed using GraphPad Prism (version 9) software, San Diego, CA, USA. Group data were compared by analysis of variance (ANOVA). For multiple comparison, the nonparametric, Tukey's post hoc test was used. Data represented as mean ± S.E.M.*p* value <0.05 is considered to be statistically significant.

## 3. Results

### 3.1. Molecular Docking and Dynamic Studies

**Figure 1:**
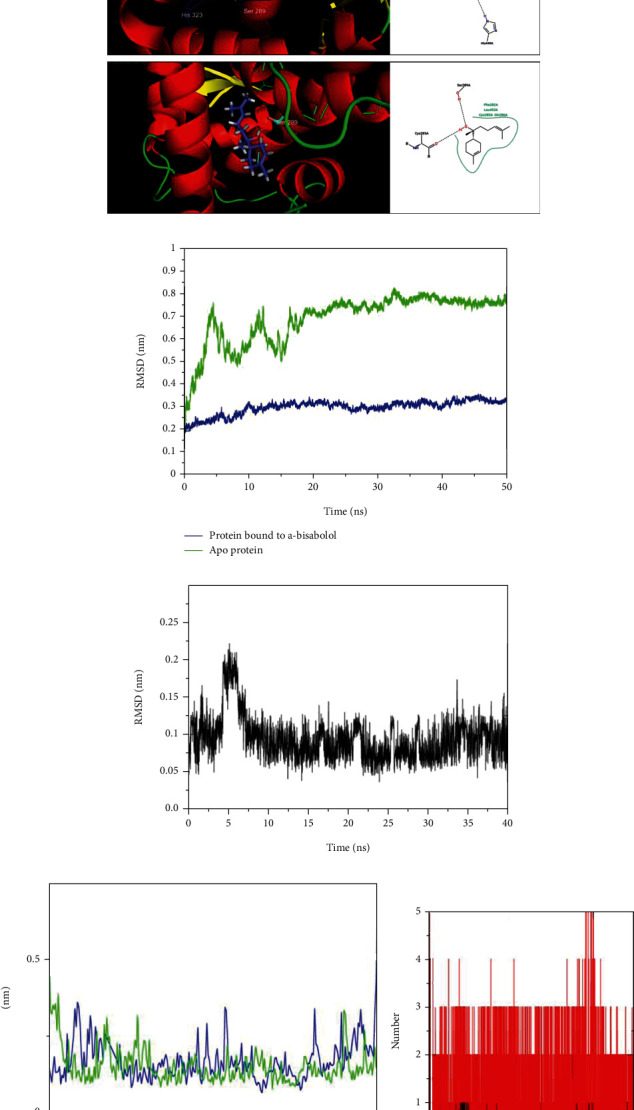
The docked pose of bisabolol and lobeglitazone and the interactions at the binding site of PPAR-*γ* and molecular dynamics studies. The docked pose of *α*-bisabolol and lobeglitazone and the interactions at the binding site of PPAR-*γ* and molecular dynamics studies. (a) Docked and cocrystallized poses of lobeglitazone are shown in blue and red stick representations, respectively. Insert picture indicates the interactions of lobeglitazone and the interactions of *α*-bisabolol at the binding site of PPAR-*γ*. (b) RMSD in protein backbone atoms, (c) RMSD in *α*-bisabolol atoms, (d) RMSF in PPAR-*γ* residues, and (e) hydrogen bond analysis for *α*-bisabolol (black lines represent hydrogen bonds, and red lines represent hydrogen bond pairs within 0.35 nm region).

The bound ligand lobeglitazone at the binding site of PPAR-*γ* was found to interact with Ser289, His323, His449, Tyr473, Cys285, Leu330, Ile341, and Met348. To validate the flexible docking protocol, the optimized structure of lobeglitazone docked at the binding site. The docked pose of lobeglitazone almost matched the cocrystallized pose, and the root means square deviation (RMSD) in the atoms in both the poses was found below 2 Å (Figure 1). The interactions produced by docked pose lobeglitazone are also similar to those produced by cocrystallized pose. This ensured the validated flexible docking protocol for further studies. The optimized structure of *α*-bisabolol docked at the binding site of PPAR-*γ* and the docking results given in the following Table 3.

### 3.2. Effect of *α*-Bisabolol on Disease Activity Index (DAI), Colon Length, and Myeloperoxidase (MPO) Activity

**Figure 2:**
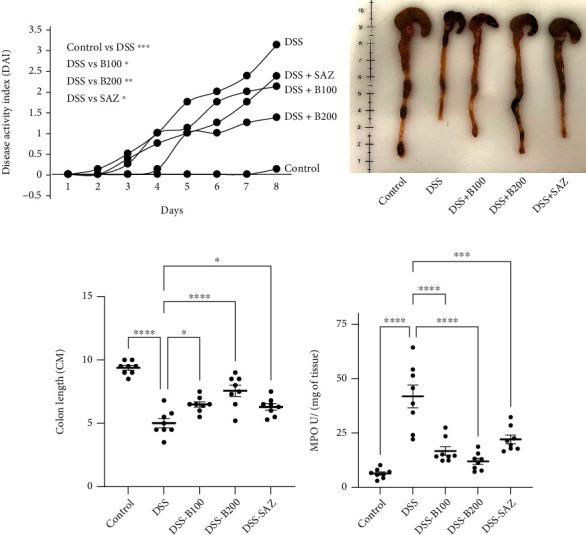
Effect of bisabolol on Disease Activity Index (DAI), colon length, and myeloperoxidase (MPO) activity. DSS administration increased DAI scores, bisabolol ,and SAZ significantly reduced OR prevented the increase in the DAI scores (a). DSS administration significantly reduced the colon length, and bisabolol and SAZ treatment prevented a decrease in colon length (b, c). DSS administration markedly increased colonic MPO activity. Bisabolol and SAZ significantly prevented the increase in MPO activity (e). Eight to ten animals were used in each group to determine statistical significance. For DAI, the statistics were performed on day eight on the cumulative DAI scores. The data expressed as mean ± SEM. ^∗^*p* < 0.05, ^∗∗^*p* < 0.01, ^∗∗∗^*p* < 0.001, and ^∗∗∗∗^*p* < 0.0001.

The DAI scores in the DSS-administered group were significantly higher compared to the control group. The treatment of *α*-bisabolol (100 and 200 mg/kg body weight) markedly decreased DAI scores in DSS-administered animals. SAZ used in treatment of IBD also decreased DAI scores; however, *α*-bisabolol effect was more potent than SAZ (Figure 2(a)). The DSS-administered group showed a marked and significant reduction in the colon length compared to the control group. Bisabolol treatment markedly prevented a reduction in the colon length in the DSS-administered group. As expected, SAZ treatment also prevented the decrease in the colon length (Figures 2(b) and 2(c)). The measurement of MPO activity serves as a surrogate marker for neutrophil infiltration in inflammation. The DSS administration significantly increased colon MPO activity compared to controls. Bisabolol treatment markedly and significantly reduced MPO activity. SAZ treatment also decreased MPO activity (Figure 2(d)).

### 3.3. Effect of *α*-Bisabolol in Colon Histology

**Figure 3:**
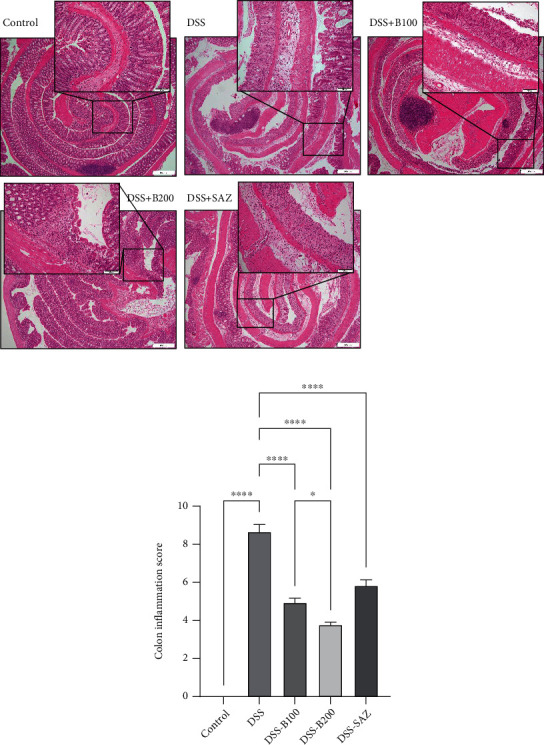
Effect of bisabolol on colon histology. The control colon microarchitecture shows healthy villi and crypts with normal submucosa and muscle layer. DSS administration resulted in focal loss of villi and crypt. The infiltration is reaching submucosa with thickening of muscle layer (a). *α*-Bisabolol treatment significantly protected the colon microarchitecture with a reduced inflammation score (b). Eight (*n* = 8) animals were used in each group to construct colon inflammation scores based on the swiss roll technique. Data are expressed as mean ± SEM. ^∗^*p* < 0.05 and ^∗∗∗∗^*p* < 0.0001.

The Swiss-roll H&E staining of control histology showed well-formed surface villi and crypts in the entire colon. The submucosa and the muscle layer thickness were normal. DSS administration resulted in the focal loss of surface epithelium, distorted villi, marked damage to the crypts, marked edema in the submucosal compartment, and thickening of the muscle layer. Bisabolol treatment markedly prevented the villi and crypt changes, decreased submucosal edema, and thickness of muscle layer compared to the group administered only DSS. This also resulted in a significant reduction in colonic inflammation scores. SAZ also prevented the changes in colonic microarchitecture and reduced colon inflammation scores. However, *α*-bisabolol was more potent in reducing colon inflammation score compared to the SAZ group (Figures 3(a) and 3(b)).

### 3.4. Effect of *α*-Bisabolol on Proinflammatory Cytokines and Mediator Protein and mRNA Expression

**Figure 4:**
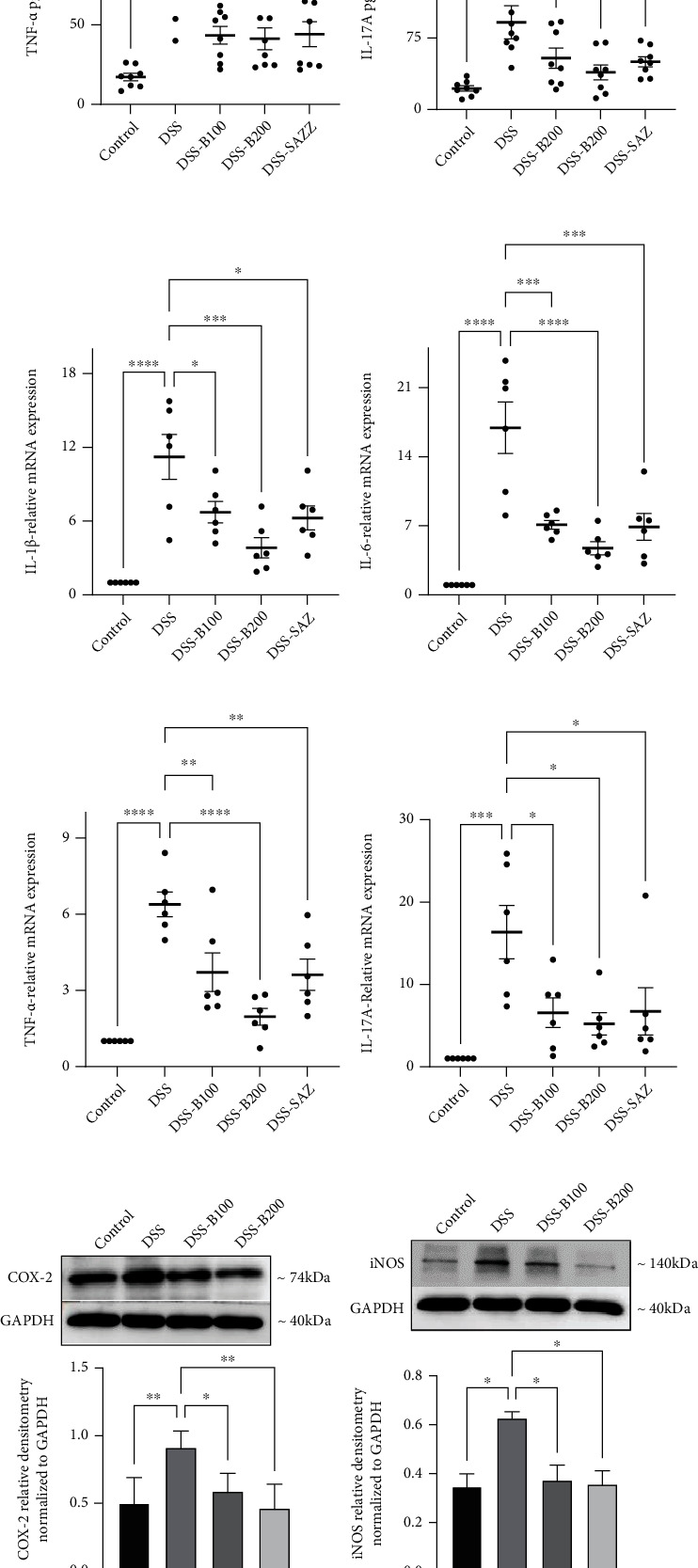
Effect of bisabolol on proinflammatory cytokines and mediator protein and mRNA expression. DSS administration significantly elevated proinflammatory cytokine concentrations. *α*-Bisabolol treatment significantly prevented the increase in proinflammatory cytokines at the protein (IL-6 (a), IL-1*β* (b), TNF-*α* (c), and IL-17A (d)) and mRNA ((IL-6 (e), IL-1*β* (f), TNF-*α* (g), and IL-17A (h)) levels. DSS administration also increased COX-2 and iNOS protein levels. *α*-Bisabolol treatment significantly downregulated protein expression (i, j). DSS administration significantly increased tissue nitrite levels, and *α*-bisabolol treatment prevented this increase (k). The density of immunoblots normalized to GAPDH. Eight (*n* = 8) animals used for ELISA, six (*n* = 6) animals used for mRNA, and six (*n* = 6) animals used for Western blot and tissue nitrite level measurements. The data is expressed as mean ± SEM. ^∗^*p* < 0.05, ^∗∗^*p* < 0.01, and ^∗∗∗^*p* < 0.001. ns: not significant.

DSS administration significantly increased expression of proinflammatory cytokine (IL-1*β*, IL-6, TNF-*α*, and IL-17A) at both the protein and mRNA levels, compared to the control group. *α*-Bisabolol (100 and 200 mg/kg body weight) treatment markedly reduced proinflammatory cytokine protein and mRNA levels in the DSS administered animals. SAZ also prevented the increase in the proinflammatory cytokines (Figures 4(a)–4(h)). The effect of *α*-bisabolol on proinflammatory mediators such as COX-2 and iNOS in DSS-administered animals was also evaluated. DSS administration significantly increased expression of COX-2 and iNOS at both the protein and mRNA levels compared to the controls. Bisabolol treatment prevented the increase in COX-2 and iNOS expression at both at protein and mRNA levels. *α*-Bisabolol treatment also prevented the DSS-induced increase in tissue nitrite levels. This correlates with the degree of iNOS expression. These results indicate that *α*-bisabolol's anti-inflammatory actions are mediated by preventing increased expression of proinflammatory cytokines and mediators (Figures 4(i)–4(k)).

### 3.5. Effect of *α*-Bisabolol on Colon MAPK, NF-*κ*B Signaling Pathways and PPAR-*γ*, PPAR-*α*, PPAR-*β*, Protein, and mRNA Expression

**Figure 5:**
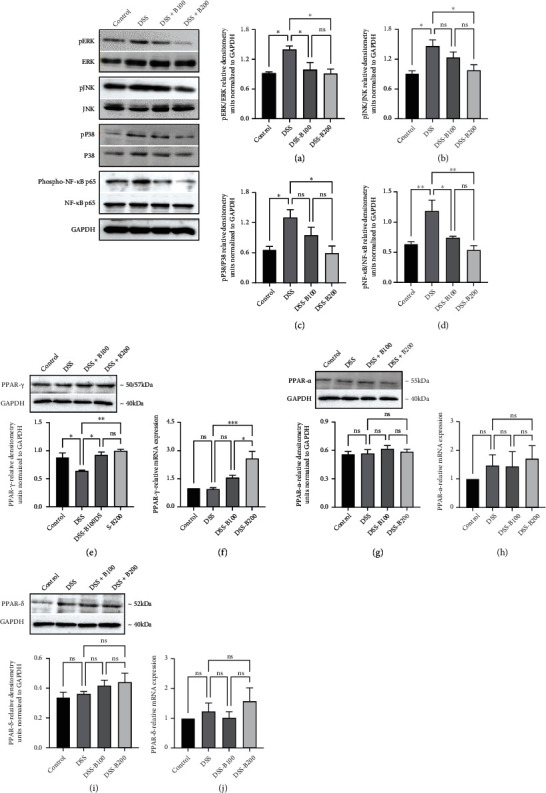
Effect of bisabolol on colon MAPK, NF-*κ*B signaling, PPAR-*γ*, PPAR-*α*, PPAR-*β*, protein, and mRNA expression. DSS administration significantly increased phosphorylation of ERK (a), JNK (b), p38 (c), and pNF-*κ*Bp65 (d) proteins. Bisabolol treatment significantly prevented the increase in the phosphorylation of these proteins. DSS administration significantly reduced expression of PPAR-*γ* at both the protein and mRNA levels. Bisabolol treatment significantly increased the PPAR-*γ* expression at both the protein and mRNA levels (e, f). PPAR-*α* (g, h) and PPAR-*β* (i, j) were not affected by DSS or bisabolol treatment at either the protein and mRNA levels. The density of immunoblots was normalized to GAPDH. Five (*n* = 5) animals used for Western blot and mRNA measurements. The data expressed as mean ± SEM. ^∗^*p* < 0.05, ^∗∗^*p* < 0.01, and ^∗∗∗^*p* < 0.001.ns: not significant.

The MAPK pathways are associated with inflammation and activate the transcription factor NF-*κ*B that regulates the expression of diverse genes involved in inflammatory pathways. Therefore, we evaluated the effect of *α*-bisabolol on the MAPK and NF-*κ*B signaling pathways in DSS-administered animals. DSS-administration significantly increased phosphorylation of the MAPK pathway proteins, ERK, JNK, and p38 in colon compared to the control group. Bisabolol treatment in DSS administered animals significantly prevented the DSS-induced phosphorylation of these proteins (Figures 5(a)–5(c)). Similarly, DSS administration increased the phosphorylation of p^ser536^NF-*κ*B p65 protein indicating its activation, while bisabolol treatment significantly reduced this phosphorylation (Figure 5(d)). PPAR-*γ* is an important transcription factor that negatively impacts colon inflammation, and it is highly expressed in the colon epithelium. Therefore, the effects of *α*-bisabolol on colon epithelial PPAR-*γ*, PPAR-*α*, and PPAR-*β* protein expression in DSS-administered animals were determined using Western blot and mRNA expression studies. DSS administration significantly downregulated PPAR-*γ* protein and mRNA expression compared to the control group. In contrast, bisabolol treatment significantly upregulated the expression of PPAR-*γ* at both the protein and mRNA levels (Figures 5(e) and 5(f)). However, PPAR-*α* and PPAR-*β* protein and mRNA expression were not affected by either DSS treatment or administration of *α*-bisabolol in DSS-administered animals (Figures 5(g)–5(j)).

### 3.6. Effect of *α*-Bisabolol Pretreatment on LPS-Stimulated RAW264.7 Macrophage Viability, Proinflammatory Cytokine Response, and PPAR-*γ*, PPAR-*α*, PPAR-*β*, and Protein Expression

**Figure 6:**
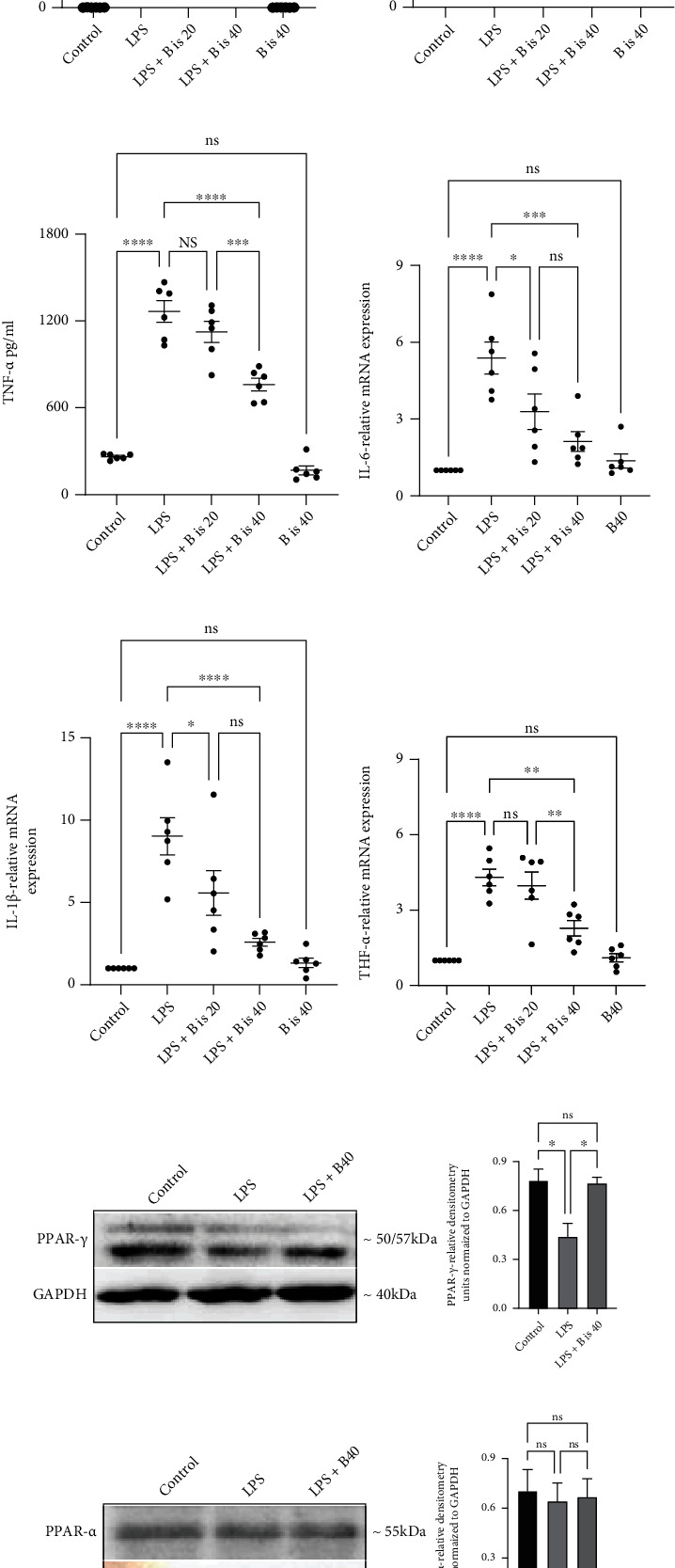
Effect of bisabolol pretreatment on LPS-stimulated RAW264.7 macrophage viability, proinflammatory cytokine response, PPAR-*γ*, PPAR-*α*, PPAR-*β*, and protein expression. Bisabolol treatment did not affect RAW264.7 cell viability at 24 h and 48 h (a, b). RAW cells were pretreated with bisabolol for 6 hr, subsequently stimulated with LPS for 24 hr. Bisabolol pretreatment significantly reduced proinflammatory cytokine response at protein (IL-6 (c), IL-1*β* (d), and TNF-*α* (e)) and mRNA levels (IL-6 (f), IL-1*β* (g), and TNF-*α* (h)). Bisabolol pretreatment significantly increased PPAR-*γ* protein expression (i),\ but did not affect PPAR-*α* (j) and PPAR-*β* (k) protein expression. Bisabolol pretreatment significantly prevented the increase in the phosphorylation of pNF-*κ*B p65 protein (l). The density of immunoblots was normalized to GAPDH. Five (*n* = 5) different experiments were run for Western blot and mRNA measurements. The data expressed as mean ± SEM. ^∗^*p* < 0.05, ^∗∗^*p* < 0.01, and ^∗∗∗^*p* < 0.001. ns: not significant.

To optimize cell treatment protocol, RAW264.7 cells were treated with various concentrations (0-100 *μ*M for 24 h and 48 h) of *α*-bisabolol to assess cell viability. No significant cell toxicity was observed even at 100 *μ*M after 24 or 48 hours of treatment (Figures 6(a) and 6(b)). Based on these initial experiments, concentrations of 20 *μ*M and 40 *μ*M *α*-bisabolol were used in subsequent investigations. Cells were pretreated with *α*-bisabolol for 6 h prior to LPS-stimulation. Subsequently, the secreted proinflammatory cytokines were determined in the cell culture supernatant, and corresponding mRNA levels were also measured using cell lysate samples. LPS stimulation significantly increased proinflammatory cytokine release (IL-1*β*, IL-6, and TNF-*α*) compared to the control. Bisabolol pretreatment significantly prevented the LPS-stimulated increases in the proinflammatory cytokine secretion and mRNA levels (Figures 6(c)–6(h)). To understand the mechanism mediating the anti-inflammatory effects of *α*-bisabolol in LPS-stimulated RAW264.7 cells, the expression of PPAR-*γ*, PPAR-*α*, PPAR-*β* proteins and phosphorylation of pNF-*κ*B p65 were carried out. We used only one dose (40 *μ*M) of *α*-bisabolol for these studies. LPS-stimulation significantly decreased the PPAR-*γ* protein expression and significantly increased the phosphorylation of the NF-*κ*B p65 transcription factor. In contrast, bisabolol pretreatment significantly increased PPAR-*γ* protein expression and decreased phosphorylation of NF-*κ*B p65. Similarly to the in vivo data, LPS-stimulation alone or after *α*-bisabolol pretreatment did not alter PPAR-*α* and PPAR-*β* protein expression (Figures 6(i)–6(l)). These results indicate that *α*-bisabolol specifically increases PPAR-*γ* transcription factor protein expression without affecting other isoforms of PPARs.

### 3.7. Effect of *α*-Bisabolol on TNF-*α* Challenged HT-29 Colonic Adenocarcinoma Cell Proinflammatory Chemokine mRNA Expression and PPAR-Gamma Promoter Assay

**Figure 7:**
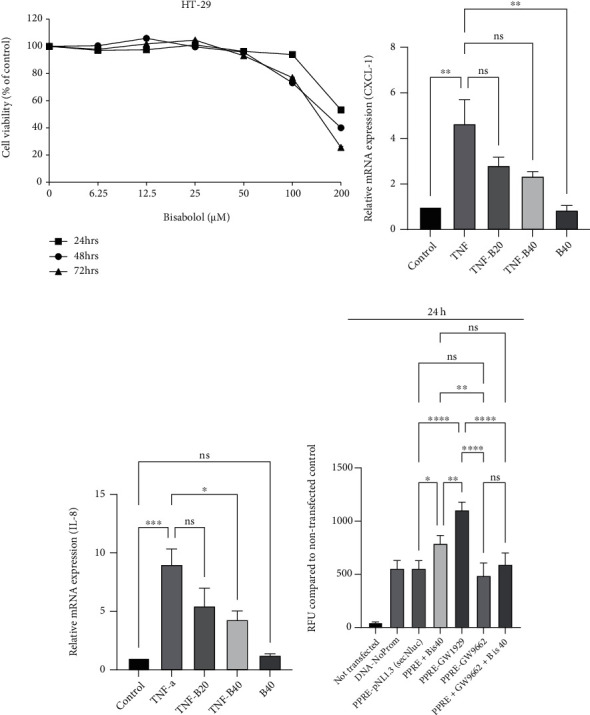
Effect of bisabolol on TNF-*α* challenged HT-29 colonic adenocarcinoma cell proinflammatory cytokine mRNA expression and PPAR-gamma promoter assay. Lower concentrations of *α*-bisabolol had no affect on HT-29 cell viability, but higher concentrations and longer time had a negative impact on cell viability (a). *α*-Bisabolol significantly decreased the expression of proinflammatory cytokine mRNA [CXCL-1 (b) and IL-8 (c)] in TNF-*α* challenged HT-29 cells. The full-length sequence of PPAR-*γ* was cloned into PPRE-pNL1.3 [secNluc] plasmid transfected using lipofectamine LTX in HT-29 cells. *α*-Bisabolol(40 *μ*M), GW1929 [PPAR*γ* agonist (1 *μ*M)], or GW9662 [PPAR*γ* antagonist (1 *μ*M)] compared to PPRE-pNL1.3 [secNluc] transfected plasmid controls. Luciferase expression at 24 h was determined in 50 *μ*L supernatant culture media using a luminometer. Four (*n* = 4) separate experiments carried out to obtain data points. The data expressed mean ± SEM. ^∗^*p* < 0.05, ^∗∗^*p* < 0.01, ^∗∗∗^*p* < 0.001, and ^∗∗∗∗^*p* < 0.0001. ns: not significant.

TNF-*α* challenged HT-29 adenocarcinoma cells is a well-accepted in vitro model system for human colon epithelial inflammation. Therefore, our initial experiments were designed to investigate the effect of various concentrations of *α*-bisabolol(0-200 *μ*M for 24 h) on HT-29 cell viability. Bisabolol was cytotoxic at the concentration of 200 *μ*M after 24 h (Figure 7(a)). Based on the cell viability assay data, we used 20 *μ*M and 40 *μ*M nontoxic concentrations for subsequent experiments. TNF-*α* caused a significant increase in expression of the proinflammatory mediators, CXCL-1, and IL-8 mRNA in HT-29 cells compared to control. Bisabolol cotreatment significantly prevented the increased mRNA expression of these inflammatory mediators (Figure 7(b) and 7(c)).

To gain further insight into whether the observed increase in the PPAR-*γ* protein expression is transcriptionally mediated, the PPAR-*γ* responsive element driving luciferase gene expression plasmid (PPRE-pNL1.3[secNluc]) was transfected into HT-29 cells to investigate the promotor response. These transfected cells were treated either with *α*-bisabolol (40 *μ*M) or with PPAR-*γ* agonist (GW1929), PPAR-*γ* antagonist (GW9662), and GW9662 + *α*-bisabolol (40 *μ*M)for 24 h period. The produced luciferase luminescence was determined spectrophotometrically from the cell culture medium. Both *α*-bisabolol and PPAR-*γ* agonist (GW1929) treatment significantly increased luciferase luminescence intensity in the culture supernatant compared to nontreated PPRE-pNL1.3 [secNluc] transfected controls. However, PPAR-*γ* antagonist treatment did not increase luminescence intensity and was similar to nontreated PPRE-pNL1.3 [secNluc] transfected controls (Figure 7(d)). These results suggest that *α*-bisabolol-mediated increase in the PPAR-*γ* protein expression is transcriptionally mediated.

## 4. Discussion

*α*-Bisabolol is a monocyclic sesquiterpene alcohol that contains one hydroxyl group, a cyclohexene ring, and a dimethyl pentenyl chain. The molecular docking and dynamic studies revealed that the propensity of key hydrogen bond formation lies only with the lone hydroxyl group, and the best-docked pose of *α*-bisabolol was found to form hydrogen bonds with Ser289 and Cys285. The lipophilic part of *α*-bisabolol occupies the hydrophobic binding cavity of PPAR-*γ* surrounded by Phe282, Leu453, Cys285, and Gln286. Although the binding energy estimate is moderately higher for *α*-bisabolol (-7.4 kcal/mol) than lobeglitazone (-9.5 kcal/mol), the molecular interaction pattern suggests that *α*-bisabolol fits well in the binding site to produce the key hydrogen bond and hydrophobic interactions. The RMSF analysis showed that the residues ranging from 380 to 476 undergo fluctuations in the case of *α*-bisabolol bound PPAR-*γ* (Figure 2(c)). Most of the residues from this region are present at the binding site, especially Ser289, Cys285, Phe282, Leu453, Gln286, Tyr473 His449, His323, Leu330, Ile341, and Met348 are important for the key nonbonded interactions. The hydrogen bond analysis also showed that *α*-bisabolol can form a maximum of two hydrogen bonds with PPAR-*γ* binding site. At least one hydrogen bond formation was consistently seen over the entire MDS (Figure 2(d)). The MDS analysis suggests that *α*-bisabolol has a strong binding affinity for the PPAR-*γ* binding site.

Based on the molecular docking and dynamics studies, to further evaluate the role of *α*-bisabolol as a PPAR-*γ* agonist in colon inflammation, both in vivo and in vitro models were employed. *α*-Bisabolol treatment significantly decreased DAI scores and maintained colon length and the microarchitecture. A similar effect of *α*-bisabolol was also observed in preserving lung histology in sepsis models of inflammation [37, 38]. DSS-induced inflammation results in increased infiltration of the immune cells which results in marked secretion of cytokine and chemokine [39]. The development of ulcerative colitis is accompanied by the activation of the NO-synthase system and the increased expression of COX-2 [40]. *α*-bisabolol significantly suppressed expression of inflammatory cytokines as well as COX-2 and iNOS at both the mRNA and protein levels. Previous studies also report a similar effect mediated by *α*-bisabolol in suppressing COX-2 and iNOS expression in LPS-induced macrophages and in a rotenone-induced rat model of Parkinson's disease [14, 41].

IL-17A is an important cytokine secreted by T-helper-17 cells in chronic inflammatory and autoimmune disease conditions. IL-17A is strongly implicated in colitis. IL-17A knockouts showed a reduced degree of inflammation than wild-type when colitis was induced using the DSS [42]. *α*-Bisabolol treatment suppressed the IL-17A expression at both the protein and mRNA levels, indicating that inhibition of the IL-17A axis is likely to contribute to the observed anti-inflammatory effects of the compound.

Activation of MAPK signaling pathways mediates the inflammatory processes. An increased expression of MAPKs has been observed in the gut mucosa of IBD patients [43, 44]. Therefore, molecules that inhibit MAPKs become an attractive target for therapy. The experimental studies of colitis carried out using specific inhibitors of JNK (inhibitor SP600125), ERK (inhibitor U0126), and p38 (inhibitor SB203580) have shown protective effects by ameliorating proinflammatory cytokine release [34, 36, 37]. *α*-Bisabolol decreased the phosphorylation of ERK, JNK, and p38 proteins involved in MAPK signaling pathways in the present study. *α*-Bisabolol has previously been shown to inhibit MAPKs in experimental models of LPS-induced lung inflammation and retinone-induced Parkinson [41, 45]. The ability of *α*-bisabolol to inhibit MAPK activation may partly explain the anti-inflammatory effects observed in our experiments.

PPAR-*γ* is highly expressed in colonic epithelial cells, as well as macrophages residing in the lamina propria adjacent to the colonic mucosa [46].Previous studies have revealed lower PPAR-*γ* expression in the colonic mucosa of UC patients compared to controls [9, 47]. Similarly, macrophages isolated from the lamina propria of DSS-induced colitis mice showed decrease PPAR-*γ* expression, indicating its importance in colon inflammation [48]. Indeed, PPAR-*γ* plays a critical role in downregulating inflammation by inhibiting NF-*κ*B activation [49, 50]. PPAR-*γ* and its ligands have been shown to inhibit macrophage activation, production of inflammatory cytokines such as TNF-*α*, IL-6, and IL-1*β*, and to downregulate NF-*κ*B signaling [3]. Our results show that *α*-bisabolol treatment specifically and significantly increased specifically PPAR-*γ* expression at protein and mRNA levels in DSS-administered animals. In contrast, the PPAR-*α* and *β*/*δ* isoforms were not affected. These findings suggest that *α*-bisabolol acts as a PPAR-*γ* ligand increasing PPAR-*γ* expression, both in the in vivo model of colon inflammation and in RAW264.7 macrophages. A previous study carried out using ethanolic extract of chamomile flowers (Matricariarecutita) showed activation of PPAR-*γ* and induction of an antidiabetic effect in mice. This has been attributed to phytoconstituents present in the ethanolic extract, including *α*-bisabolol [51, 52].

HT-29 adenocarcinoma cells exhibit several cellular aspects of human colonic epithelial cells [53]. Therefore, our next aim was to investigate whether *α*-bisabolol mediates anti-inflammatory effects in HT-29 cells. To mimic the inflammatory status, HT-29 cells were challenged with TNF-*α*, a well-established in vitro model of colon inflammation [54]. The HT-29 cell viability assay indicated that *α*-bisabolol showed cellular toxicity at high concentrations (100 *μ*M or higher). Based on these results, we selected concentrations of 20 and 40 *μ*M, which did not affect cell viability. *α*-Bisabolol significantly decreased CXCL-1 and IL-8 chemokine mRNA expression in TNF-*α* challenged HT-29 cells, further supporting its potent anti-inflammatory effect.

PPAR-*γ* belongs to the nuclear receptor family consisting of approximately 50 transcription factors implicated in many biological functions. It is an essential nuclear receptor controlling the expression of many regulatory genes in lipid metabolisms, insulin sensitization, inflammation, and cell proliferation [55]. PPAR-*γ* activation occurs through ligand binding that leads to a conformational change in the receptor, allowing the recruitment of coactivator proteins to induce transcriptional activation. The transcriptional activity of PPAR-*γ* is regulated by posttranslational changes such as phosphorylation or ubiquitination. The activation requires heterodimerization within the nucleus with another nuclear receptor named retinoid X receptor *α* (RXR*α*), leading to bind a specific DNA sequence elements known as peroxisome proliferator elements (PPREs) [56]. Many dietary nutrients and phytochemicals modulate PPAR-*γ* activity by activating the receptors [57]. However, a few studies have also shown an increase in the PPAR-*γ* protein expression apart from receptor activation. In the current study, we have also observed an increase in the PPAR-*γ* protein expression. Similar effects have also been seen with other PPAR-*γ* agonists in studies from other groups [58, 59]. The PPAR-*γ* agonist LYSO-7 prevented inflammation and reduced neutrophil infiltration and myeloperoxidase activity in an ethanol/HCl-induced gastric lesion model in rats. LYSO-7 mediated the anti-inflammatory effect by increasing the expression of PPAR-*γ* at both the mRNA and protein levels [59]. In a model where the intraluminal application of acetic acid resulted in chronic gastric ulcers in rats, this was accompanied by elevation of proinflammatory cytokines (TNF-*α* and IL-1*β*) and protein levels of the nuclear p65 subunit of NF-*κ*B, but decreased the PPAR-*γ* gene expression [58]. In this model, the PPAR-*γ* ligand, pioglitazone reduced the severity of ulceration and repressed levels of TNF-*α* and IL-1*β* and the nuclear p65 subunit, but in contrast, increased the abundance of PPAR-*γ* in the gastric mucosa [58]. In another study, a *β*-carboline alkaloid, harmine increased expression of PPAR-*γ* through a mechanism involving the Wnt signaling pathway, but it did not directly activate PPAR-*γ* in transactivation analyses [60].

The observed increase in the PPAR-*γ* expression mediated by *α*-bisabolol in the DSS-administered colitis model and in RAW264.7 macrophages could be transcriptionally mediated. Therefore, to address this, HT-29 adenocarcinoma cells transfected with PPRE-pNL1.3 plasmid containing PPAR-*γ* promoter were treated 40 *μ*M concentration of *α*-bisabolol [61]. The *α*-bisabolol treatment significantly increased PPAR-*γ* promoter-driven luciferase expression. The *α*-bisabolol mediated promoter expression was comparable to that of the PPAR-*γ* agonist GW1929.These results indicate that the increased PPAR-*γ* expression by *α*-bisabolol is transcriptionally regulated.

## 5. Conclusions

Phytochemical-based natural ligands present in medicinal plants are of interest in inflammatory bowel disease drug discovery, because of their potential to increase expression and activation of PPAR-*γ* in the colon. Our studies have confirmed that *α*-bisabolol has potent anti-inflammatory properties to mitigate colonic inflammation. The observed anti-inflammatory activities mediated by *α*-bisabolol are mainly attributed to increased colon PPAR-*γ* expression and suppression of MAPK, inflammatory cytokine, and NF-*κ*B signaling pathways. *α*-Bisabolol or its analogues may be valuable for the treatment of inflammatory bowel disease as well as other inflammatory conditions.

## Figures and Tables

**Table 1:** Disease Activity Index Score.

Disease Activity Index Score calculation weight loss	Score	Stool consistency	Score	Rectal bleeding
No loss	0	Normal	0	No blood
1-5%	1	Mild soft	2	Heme occult +ve& visual brown fecal pellet
5-10%	2	Very soft	3	Heme occult +ve& visual red color fecal pellet
10-20%	3	Diarrhea	4	Gross bleeding and blood around anus
>20%	4			

**Table 2:** Determination of colon inflammation score.

Colon inflammation grade	Percentage of inflammation involvement of mucosal surface area
0: none	0: no inflammation
1: mild	1: 1-25%
2: moderate	2: 26-50%
3: severe	3: 51-75%

**Table 3:** Docking results.

Compound	Binding free energy estimate (kcal/Mol)	H-bond interactions	Hydrophobic interactions
*α*-Bisabolol	-7.4	Ser289, Cys285	Phe282, Leu453, Cys285, Gln286
Lobeglitazone	-9.5	Ser289, Tyr473, His449, His323	Cys285, Leu330, Ile341, Met348

## Data Availability

The data availability can be requested from the corresponding author.
